# Analysis of real-world data on growth hormone therapy adherence using a connected injection device

**DOI:** 10.1186/s12911-020-01183-1

**Published:** 2020-07-29

**Authors:** Ekaterina Koledova, Vincenzo Tornincasa, Paula van Dommelen

**Affiliations:** 1 Endocrinology Global Medical, Safety and CMO, Merck KGaA, 64293 Darmstadt, Germany; 2 Merck Connected Health and Devices, Ares Trading S.A., an affiliate of Merck KGaA, 1262 Eysins, Switzerland; 3 The Netherlands Organization for Applied Scientific Research TNO, Leiden, The Netherlands

**Keywords:** Adherence, eHealth, Electromechanical injection device, Growth disorders, Growth hormone, Population health

## Abstract

**Background:**

Poor adherence to long-term recombinant human growth hormone (r-hGH) treatment can lead to suboptimal clinical outcomes; consequently, supporting and monitoring adherence is a crucial part of patient management. We assessed adherence to r-hGH treatment in children with growth disorders over 48 months using a connected monitoring device (easypod™), which automatically transmits adherence data via an online portal (easypod™ connect); both sit within an adherence decision support system (ADSS). We also investigated the effect of age and sex on adherence.

**Methods:**

Data from children transmitting over 10 injections between January 2007 and February 2019 were analyzed. Adherence (mg injected/mg prescribed) was categorized as high (≥85%), intermediate (> 56–84%) or low (≤56%) and assessed at seven time points from the start of treatment up to 48 months. Adherence was investigated over time and stratified by puberty status and sex. Mean transmission rate in each adherence category (total number of transmissions/total number of children) at each time point was calculated as a proxy measure of engagement in disease and treatment management. Descriptive analyses were performed.

**Results:**

Longitudinal records were available for 13,553 children. Overall, 71% (*n* = 9578) had high adherence, 22% (*n* = 2989) intermediate and 7% (*n* = 986) low. The proportion of children with high adherence decreased over time from 87% (*n* = 12,964) to 65% (*n* = 957) and was higher in pre-pubertal than pubertal children (girls: 80% [*n* = 1270] vs 70% [*n* = 4496]; boys 79% [*n* = 2573] vs 65% [*n* = 5214]). Children with high adherence had a higher mean number of transmissions (12.5 [SD 24.9]) than children with intermediate (7.2 [SD 15.3]) or low (3.5 [SD 5.7]) adherence.

**Conclusions:**

High adherence was seen in patients administering r-hGH using the connected device. Children with high adherence were most likely to regularly transmit data. Pubertal children showed lower adherence. We show the potential to develop an ADSS to analyze trends in real-world adherence data. This may prove useful to direct interventions to improve adherence while the ability to readily share data with healthcare professionals may itself improve adherence.

## Background

Recombinant human growth hormone (r-hGH) is used in the treatment of various growth disorders [1]. Suboptimal final growth and poor clinical outcomes [2] can result from poor adherence due to factors such as complex drug regimens, limited access to care and issues in puberty [3, 4].

Traditional approaches to monitoring adherence such as patient testimony, or proxy measurements, such as prescription records or vial counting [5], are not sufficiently objective. New eHealth-based ecosystems with automatic adherence recording and data transmission allow real-time monitoring of adherence. By alerting healthcare professionals (HCPs) to take action if a decrease in adherence is detected, these ecosystems can provide personalized individual-patient or targeted support for groups of patients at risk of low adherence, thereby contributing to positive population-health outcomes [6, 7].

Automatic recording and monitoring of adherence is also important in the development of treatment plans. Innovative eHealth solutions and technologies, as part of an adherence decision support system (ADSS), offer the potential to improve health outcomes by seamlessly tracking adherence according to treatment plans and engaging and informing patients and HCPs through patient support programs (PSPs) [8]. ADSS is a form of clinical decision support that provides HCPs, patients and care providers with timely knowledge and person-specific information, to improve health care and outcomes [9]. Many existing technological interventions aim to address non-adherence to medication but not all deliver the full potential of an ADSS [10–13]. For example, by using an ADSS, the sharing of prospective adherence tracking data with HCPs ahead of scheduled clinic visits may enable HCPs to detect both “true” poor responders to r-hGH treatment and those patients whose poor response is due to suboptimal adherence. The use of mobile phone medical applications is common and has been associated with improved adherence and outcomes in areas such as exercise [14, 15].

Applications also exist for growth monitoring, although these lack integration with a connected drug-delivery device that removes the bias associated with self-reported patient outcomes [16, 17]. The ability of electronic devices and everyday objects to communicate, interact, be remotely controlled and monitored by other devices through internet connectivity is known as the ‘internet of things’ (IoT) [18–20]. Advancements in IoT-based applications in the healthcare domain has facilitated collection of real-world data from a wide range of connected devices, such as pill dispensers using medication event monitoring [21–23] and pen devices for the treatment of diabetes [24, 25]. However, there are few IoT-based solutions for the monitoring and understanding of adherence in other endocrine conditions requiring injectable treatments, where adherence monitoring is key to ensuring optimal care. A prime example is the gap in health technology and informatics for growth hormone deficiency, which requires injectable treatment over long periods of time in a mostly pediatric population [26].

**Fig. 1:**
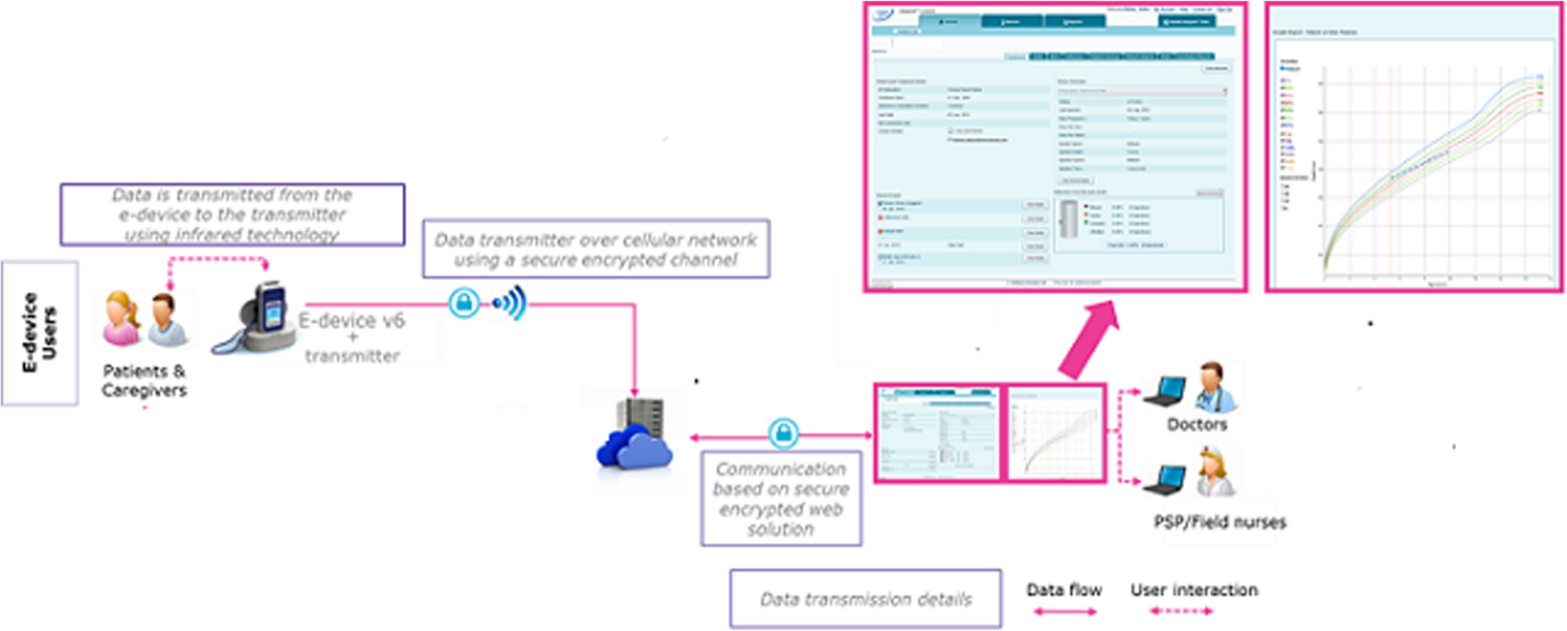
The easypod™ connect ecosystem

The easypod™ injection device was developed for patients with growth hormone deficiency, to meet the requirements for a reliable, easily used, convenient injection device with IoT functionality [27]. It is the first IoT-based connected device to electronically record adherence data (completed and missed doses) for patients receiving r-hGH (Saizen®, Merck KGaA, Darmstadt, Germany) to treat growth disorders. HCPs can access adherence data via the easypod™ connect ecosystem [28]. The easypod™ connect transmitter can transfer the injection data recorded by the easypod™ to a cloud-based platform using a cellular connection. The data are then accessible to HCPS through the dedicated web-based dashboard or to patients from a mobile application (Fig. 1). Based on the adherence data, HCPs can counsel patients and adjust the dosage so the connected device ecosystem meets the full potential of an ADSS.

The connected device is currently approved for use in more than 40 countries [29]. The observational ECOS study has reported on the robust adherence data that has been collected using the online portal. Most patients maintained an adherence rate of ≥80% for > 3 years, with poor adherence linked to suboptimal clinical outcomes [28, 30]. Such data on adherence and engagement with the device in a real-world setting have only recently been available.

Our aim was to perform a real-world observational analysis of the connected device ecosystem to gather insights about recorded adherence and engagement to treatment in children with growth disorders, from the start of treatment up to 48 months. We also investigated the effect of age and sex on adherence.

## Methods

Treatment with the connected device and connect platform was conducted according to local practice. The study employed inclusion/exclusion criteria and an approach to treatment and overall care that was representative of the clinical practice for each of the countries enrolled. Prior to their participation in the real-world, observational, retrospective analysis for monitoring r-hGH adherence and outcomes, both parents of the pediatric patients or the adult patients reviewed and voluntarily signed an informed consent form materializing their agreement for data collection, storage and use of their pseudonymized data to create aggregated statistical and general adherence reports.

### Data transmitted to an internet cloud

The connected device sits within an ecosystem, through which injection data are transmitted to a secure cloud on the internet via a home station (Fig. 1). This ecosystem was used to collect longitudinal data on > 13,553 patients to study adherence patterns over a period of 13 years. Data from children transmitting > 10 injections between January 2007 and February 2019 were analyzed. For the descriptive statistics at each time point, we included the children who were still on treatment at that moment in time.

### Definition and grades of adherence

Adherence (mg injected/mg prescribed) was categorized as high (≥85%), intermediate (> 56–84%) or low (≤56%) [2] and assessed at seven time points. High adherence was defined as missing no more than one dose a week on average. Adherence was then explored by puberty status (nominal cut-offs at 10 years for girls and 12 years for boys) and sex. Dosage and frequency as per the connected device settings were defined by the HCPs.

Data transmissions were instigated by the child, guardian or by HCPs on behalf of the patient at each clinic visit. Adherence was recorded for children transmitting data at each time point. No imputation was made for missing data or withdrawal. For each adherence cohort, the mean number of transmissions (total number of transmissions in each adherence category versus the total number of children in each adherence category) was calculated as a proxy measure of engagement in disease management.

### Statistical analysis

Descriptive statistics were used to describe differences over time in adherence (low/intermediate/high), by puberty status (pre-pubertal/pubertal) and sex, and overall adherence by transmission ratio.

## Results

**Fig. 2:**
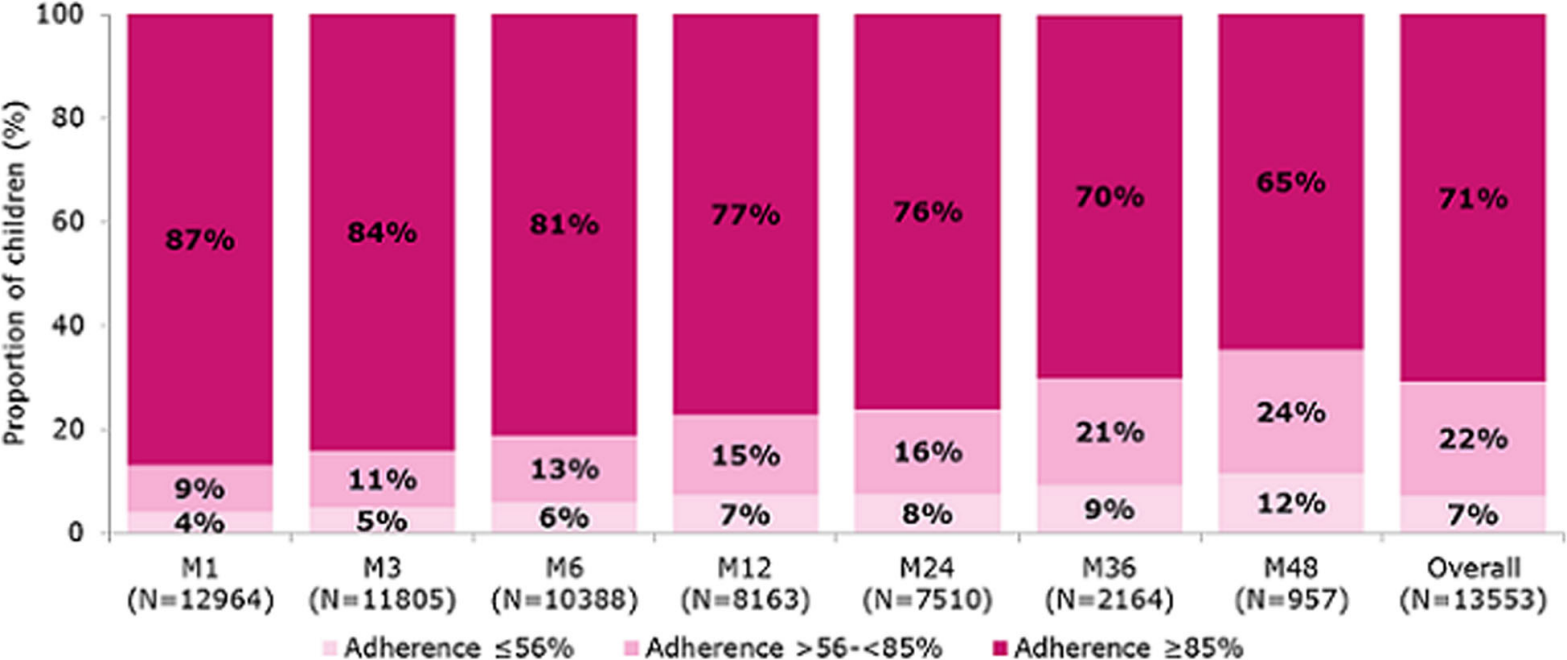
Proportion of children in each adherence category at each time point. M, month

Longitudinal records were available for 13,553 children between January 2007 and February 2019. Different adherence patterns arose from the data analysis. Overall, more children had high adherence (*n* = 9578) than intermediate (*n* = 2989) or low (*n* = 986) adherence. The proportion of children with high adherence decreased from 87% (*n* = 12,964) to 65% (n = 957) between months 1 and 48; however, at each time point up to month 48, more children had high adherence than low/intermediate adherence (Fig. 2).

Compared with children in the low/intermediate adherence categories, children with high adherence also had the highest mean number of transmissions (Table 1).

**Table 1:** Overall demographics according to adherence rate

	Adherence ≥ 85% (*n =* 9578)	Adherence > 56% to < 85% (*n =* 2989)	Adherence ≤ 56% (*n =* 986)
Boys, mean age (SD)	12.8 (5.3 years)	15.0 (6.6) years	15.3 (7.6) years
Number of boys < 12 years/12+ years	2026/3377	417/1384	130/453
Girls, mean age (SD)	12.2 (5.2) years	14.3 (7.9) years	15.8 (10.6) years
Number of girls < 10 years/10+ years	1018/3157	195/993	57/346
Mean (SD) number of transmissions	12.5 (24.9)	7.2 (15.3)	3.5 (5.7)
Mean (SD) global adherence duration, days	599 (530)	694 (565)	630 (539)

**Fig. 3:**
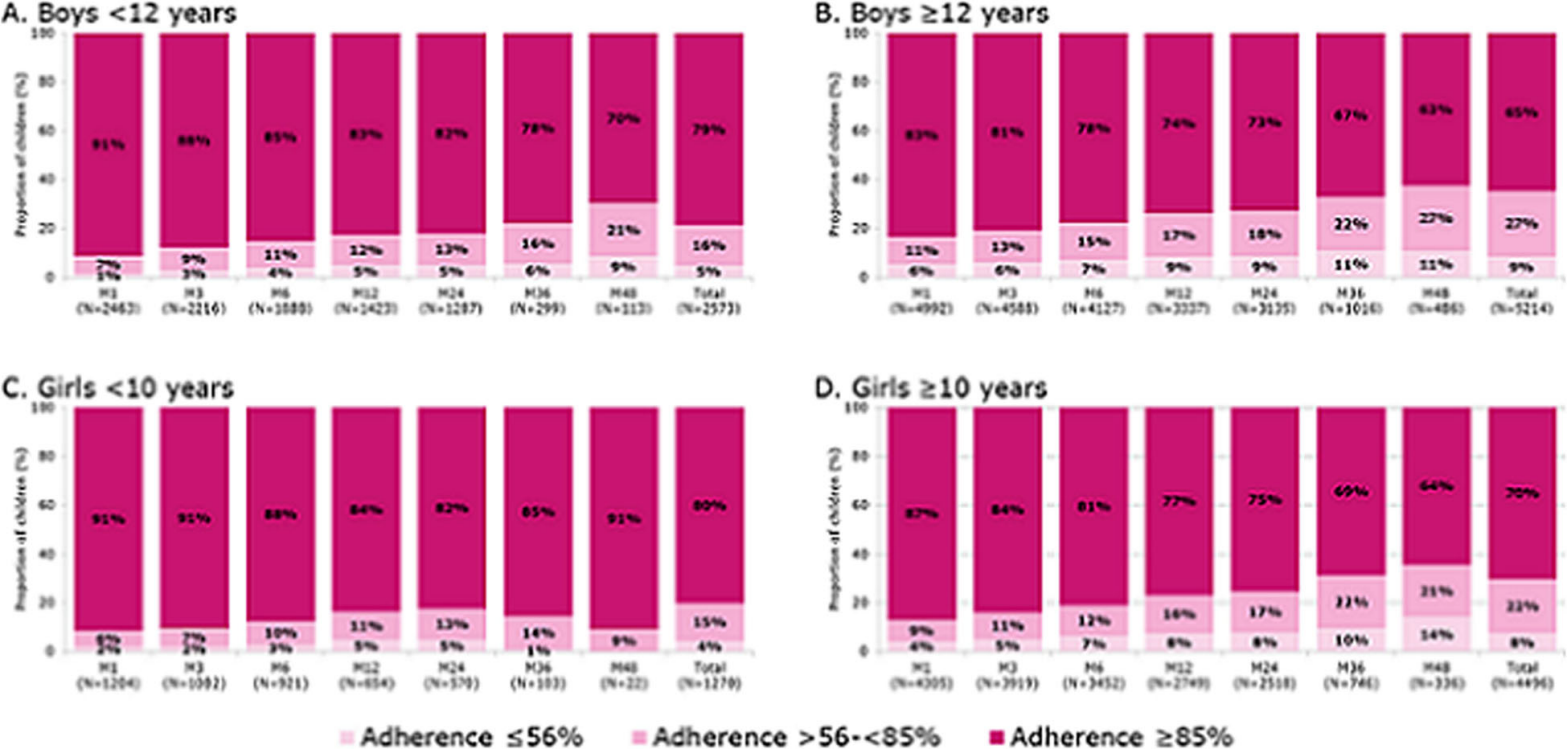
Proportions of children adherent at each time point according to age and sex. M, month

The proportion of children with high adherence overall was slightly higher in girls than in boys (72% [*N* = 5766]) vs 69% [*N* = 7787]); a similar trend was seen at each time point. The proportion of pre-pubertal girls with high adherence was greater than the proportion of pubertal girls (80% [*N* = 1270] vs 70% [*N* = 4496]). Similar results were found in boys (79% [*N* = 2573] vs 65% [*N* = 5214]) (Fig. 3).

## Discussion

We showed the distribution of adherence by age and sex in a large, real-world setting up to 48 months after the start of treatment and concluded that the majority of children who receive r-hGH treatment via the connected device had high adherence. A higher proportion of pre-pubertal than pubertal children and a slightly higher proportion of girls than boys were in the high adherence category. The connected device was a useful tool in this regard as it is unique in providing the most objective measurement of patient adherence to r-hGH that is currently available. Monitoring using the connected device is based on the real-time recording of the doses administered compared with those prescribed, rather than incidental or proxy recording methods. Patients using ostensibly objective methods of monitoring can still substantially overestimate their level of adherence to r-hGH treatment, owing to a combination of factors [31].

There was a trend for the children who were most adherent to treatment to be more likely to transmit their injection data results regularly than those who were less adherent. This association between adherence and transmission of data may indicate that sharing data with HCPs impacts adherence rates. The active engagement of patients and caregivers through the use of eHealth solutions is already known to improve treatment adherence in patients with chronic conditions and is associated with long-term improvement in clinical outcomes [31]. Data from wearable and monitoring devices are widely used in observational studies [32, 33]; however, the reporting of adherence data is limited and small scale [10–13, 26]. This analysis shows the value of large-scale real-world datasets using connected devices, in particular for monitoring hormone treatment adherence.

The results reported here may suggest that pubertal children are at greater risk for suboptimal adherence and may benefit from closer engagement. Real-time monitoring using internet connected devices has the potential to benefit both the patient and the physician and may provide a starting point for discussions on any issues the patient may have encountered. Alternatively, the output can be used proactively on a target group or population level, to identify times or define targets when patients may need particular attention. Integrating a holistic approach to patient care such as patient-reported outcome measures (PROMs) and patient-reported experience measures (PREMs) may improve understanding of non-adherence and inform PSPs [34]. To develop appropriate and succinct PROMs and PREMS, human factor studies on drivers for adherence to hGH would be beneficial [35].

Electronic injection reminders are a feature of the easypod™ device, however adherence may be further enhanced through mobile phone reminders and applications [36–38]. Gamified interventions for pediatric patients are receiving increasing attention as a potential tool to promote medication adherence through factors such as goal setting, incentive-based engagement and education [38]. Although limited, there is evidence suggesting positive health and behavioral outcomes associated with gamification in pediatric patients [39–41]. Design frameworks are lacking for guiding the development of gamified health interventions and further research is needed within this area to optimize the influence of applications on self-management [42]. To complement the use of the easypod™ device, a treatment-support application was recently launched in Hong Kong [43]. It is an educational tool that incorporates elements of gaming to encourage accurate r-hGH administration. The application features an avatar; studies have suggested that use of an avatar can serve as a role model and influence positive behaviors [44]. The impact of this gamified intervention and its potential on a wider scale is yet to be determined.

The advantages of this real-world dataset are the large population base, multinational setting, automated recording of adherence and injection settings and manually entered basic patient characteristics (such as age and sex), and the potential to develop a complete ADSS. The results of this analysis are highly aligned with real-world practice and corroborate individual case studies of children using the connected device, which show that direct monitoring of adherence can affect motivation and management [6, 45, 46].

However, the dataset has limitations that are inherent in any real-world datasets. For example, there may be missing information (e.g., owing to a lack of transmission) or there may be high inter-patient variability (e.g., owing to changes in the treatment plan or other actions taken by the HCP or child). In addition, many confounding, contextual or patient-specific variables that could influence behavior beyond sex and age are not recorded automatically and therefore were not available for this analysis. Although this study reflects a large amount of data, it should also be considered that data collection practices may differ between countries, which makes such comparisons challenging.

Future opportunities for cross-linking the real-world dataset with electronic medical records (EMRs) or electronic health record (EHR) datasets would make the assessment of outcomes much more insightful, given the greater availability of treatment plans and outcome data now available. Recent developments in computing interfaces focused on supporting seamless data transfer demonstrate potential for approaching integration into EMRs and EHRs [47]. However, direct integration of patient data presents additional challenges such as legal and ethical considerations [48] and the fragmentation of the technological landscape, in which each country appears to have its own specific subset of EHR systems and vendor which are specific to local practice.

## Conclusions

These results show the potential of using a connected device to study patterns of adherence across large populations. Our real-world data suggest that high adherence is seen in children using the connected device ecosystem for treatment of growth disorders with r-hGH, although the proportion of children with high adherence decreased over time. More pre-pubertal than pubertal children and slightly more girls than boys had high adherence. Children who were most engaged with the device were more likely to have better adherence. The use of the connected device ecosystem combined with EHRs has the potential to realize an ADSS that can facilitate the assessment of outcomes and adherence plans to support integrated care.

## Data Availability

The datasets generated and/or analyzed during the current study are not publicly available due to the dataset containing information derived from personal data, which could be aggregated to a degree that could potentially identify individual subjects but are available from the corresponding author on reasonable request. For all new products or new indications approved in both the European Union and the USA after 1 January, 2014, Merck KGaA (Darmstadt, Germany) will share patient- and study-level data after deidentification, as well as redacted study protocols and clinical study reports from clinical trials in patients. These data will be shared with qualified scientific and medical researchers, upon a researcher’s request, as necessary for conducting legitimate research. Such requests must be submitted in writing to the company’s data sharing portal and will be internally reviewed regarding criteria for researcher qualifications and legitimacy of the research purpose.

## Abbreviations

ADSS: Adherence decision support system
EHR: Electronic health record
EMR: Electronic medical record
GH: Growth hormone
HCP: Healthcare professional
IoT: Internet of things
PREM: Patient-reported experience measure
PROM: Patient-reported outcome measure
PSP: Patient support program
r-hGH: Recombinant human growth hormone
